# Cortical metabolic and structural differences in patients with chronic migraine. An exploratory ^18^FDG-PET and MRI study

**DOI:** 10.1186/s10194-021-01289-5

**Published:** 2021-07-17

**Authors:** Marta Torres-Ferrus, Deborah Pareto, Victor J Gallardo, Gemma Cuberas-Borrós, Alicia Alpuente, Edoardo Caronna, Adrià Vila-Balló, Carles Lorenzo-Bosquet, Joan Castell-Conesa, Alex Rovira, Patricia Pozo-Rosich

**Affiliations:** 1 Headache and Craniofacial Pain Unit, Neurology Department, Vall d’Hebron University Hospital, Barcelona, Spain; 2 Headache and Neurological Pain Research Group, Vall d’Hebron Research Institute (VHIR), Universitat Autònoma de Barcelona, Barcelona, Spain; 3 Section of Neuroradiology, Department of Radiology, Vall d’Hebron University Hospital and Research Institute (VHIR), Barcelona, Spain; 4 Research and Innovation Unit , Althaia Xarxa Assistencial Universitària de Manresa , Manresa, Spain; 5 Nuclear Medicine Department , Vall d’Hebron University Hospital, Barcelona, Spain

**Keywords:** Chronic migraine, Neuroimaging, Positron emission tomography, Cortical thickness, Metabolism, Structural

## Abstract

**Background:**

To describe interictal brain structural and metabolic differences between patients with episodic migraine (EM), chronic migraine (CM) and healthy controls (HC).

**Methods:**

This is an exploratory study including right-handed age-matched women with EM, CM and HC. On the same day, a sequential interictal scan was performed with ^18^FDG-PET and MRI. 3D T1-weighted images were segmented with FreeSurfer, normalized to a reference atlas and the mean values of metabolism, cortical thickness (CTh) and local gyrification index (IGI) were determined. Groups were compared using age-adjusted linear models, corrected for multiple comparisons. ^18^FDG-PET measurements between groups were also analysed adjusting by patient’s age, CTh and lGI. The variables independently associated with diagnosis were obtained using a logistic regression analysis.

**Results:**

Fifteen patients (8 EM, 7 CM) and 11 HC were included. Morphometric data showed an increased CTh in 6 frontal areas (L/R-Caudal Middle Frontal, L/R-Rostral Middle Frontal, L-Medial Orbitofrontal and L-Superior Frontal) in CM patients compared to HC without differences for IGI. The structural adjusted analysis in CM showed a statistically significantly hypometabolism in 9 frontal areas (L-Lateral Orbitofrontal, L/R-Medial Orbitofrontal, L-Frontal Superior, R-Frontal pole, R-Parts Triangularis, L/R-Paracentral and R-Precentral) and 7 temporal areas (L/R-Insula, L/R-Inferior temporal, L/R-Temporal pole and R-Banks superior temporal sulcus) compared to HC. EM patients presented intermediate metabolic values ​​between EM and HC (non-significant).

**Conclusions:**

CM patients showed frontotemporal hypometabolism and increased frontal cortical thickness when compared to HC that may explain some cognitive and behavioural pain-processing and sensory integration alterations in CM patients. Combined information from sequential or simultaneous PET and MRI could optimize the study of complex functional neurological disorders such as migraine.

**Supplementary Information:**

The online version contains supplementary material available at 10.1186/s10194-021-01289-5.

## Background

Migraine is a complex chronic neurological disorder that is characterized by the presence of recurrent attacks of headache and other neurological symptoms. Migraine is thought to conform a disease spectrum with symptoms and headache frequency gradually increasing in some patients, evolving from an episodic to a chronic form [1]. This transformation, is dynamic and characterized by several progressive neurophysiological and neuroanatomical changes in pain, sensory or affective-related brain areas [2, 3].

Advanced neuroimaging could help us to better understand which brain structures are involved in migraine pathophysiology as well as to find potentially useful migraine imaging biomarkers [3]. Although there is an increasing number of neuroimaging studies in migraine, only a few of them have specifically included chronic migraine (CM) patients. Previous MRI studies have highlighted that brain regions with a key role in migraine attack generation, like the pons and hypothalamus might also be involved in migraine chronification [4–8]. Besides, non-controlled presence of comorbidities or medication as well as comparisons with true non-headache controls, make it difficult to draw accurate conclusions.

18 F-fluorodeoxyglucose positron emission tomography (^18^ F-FDG-PET) is used to produce images of metabolic activity of the brain. Increased glucose uptake may be due to the presence of a hyperactive or dysfunctional brain state as reduced 18 F-FDG uptake has been observed in abnormally functioning regions of brain in numerous disorders including dementia, stroke or interictally epilepsy [9]. Specifically in migraine, ^18^ F-FDG-PET studies have identified disease or attack-specific alterations in brain activity within the migraine brain [10]. However, ^18^ F-FDG-PET imaging can only offer relatively poor anatomical information while MRI is considered to be a precise structural imaging modality.

The aim of this study was to describe interictal brain metabolic and structural differences in a controlled and homogeneous population of patients with episodic migraine (EM), CM and healthy controls (HC). Moreover, we analyse how structural brain differences between participants can influence their cerebral activity in an exploratory analysis.

## Methods

### Subjects

This was a case-control study. We included right-handed adult women, 18 to 60 years of age, with EM and CM fulfilling International Headache Disorders Classification – 3beta [11] that were not taking any preventive treatment for migraine and had never previously been on one. We included a group of age-matched healthy women as control group. We excluded patients with neurological or psychiatric comorbidities as well as other chronic pain syndromes, or patients with incidental relevant structural or metabolic abnormalities on imaging studies. Patients who were taking medications with central nervous effect or drugs used as migraine preventive for other indications (including antidepressants, neuroleptics benzodiazepines, antiepileptic, betablockers, antihypertensive medications and OnabotulinumtoxinA) and patients with analgesic overuse were also excluded. Inclusion period went from October 2016 to January 2018.

All participants were evaluated and diagnosed by a headache specialist using headache diaries. HC were also evaluated in depth, in order to rule out presence of any type of recurrent headaches or history of migraine in a first degree relative. We collected clinical and demographical data. All participants were screened for anxiety and depression with Spanish version of State-Trait Anxiety Inventory (STAI) [12] and Beck Depression Inventory II (BDI-II) [13] and completed a health status evaluation with 36-items Short Form Health Survey (SF-36) [14] and Perceived Stress Scale [15]. Migraine participants also completed an evaluation of headache-related disability with Spanish version of Migraine Disability Assessment (MIDAS) [16], Headache Impact Test 6 (HIT-6) [17] and Migraine-Specific Quality of Life Questionnaire version 2.1 (MSQ) [18] and were asked to complete a daily headache diary for, at least, a month before the imaging protocol.

### Neuroimaging protocol

Interictal brain ^18^ F-FDG-PET and MRI were performed on the same day. Patients who had a migraine attack the day of the exam were rescheduled. Since CM patients may have not had headache-free days, we did not exclude patients with mild headache. Patients were asked to rate headache pain (0–10 Numerical Scale) before the scan. ^18^ F-FDG-PET was performed with 6 h fasting and blood glucose levels < 150 mg/dL, and MRI was performed 6 h after the ^18^ F-FDG-PET to minimize radiation exposure during the procedure.

^***18***^*** F-FDG-PET acquisition.*** 150 MBq of 18 F-FDG was injected intravenously, and imaging was performed using a fusion PET-computed tomography (CT) scanner (Siemens Biograph mCT64S) after 30 min. The CT scan was used for attenuation correction. The scanning parameters may vary according to the type of CT scanner. Usually, the tube voltage is set around 120-140 kV. Brain PET acquisition started 30 min in 3-D mode. Brain PET image reconstruction utilized an iterative algorithm (4 iterations, 12 subsets) with a 128 × 128 matrix size, 3.18mm pixel size and a post-filter Gaussian of 4 mm, TOF, ultra HD-PET, decay and attenuation correction. Prior to the administration of 18 F-FDG, a blood glucose determination was performed, in case of hyperglycemia (> 160 mg/dl), due to competitiveness with plasma levels and FDG, it was postponed until the blood glucose levels were normal.

***MRI acquisition.*** MRI images were acquired in a 3.0T magnet (Tim Trio, Siemens, Erlangen, Germany) with a 12-channel phased-array head coil. The protocol included, among other sequences, a sagittal 3D T1-weighted MPRAGE (Magnetization Prepared – Rapid Gradient Echo) (TR = 2300ms, TE = 2.98ms, FOV = 256 × 256mm, 192 sections, voxel size = 1 × 1 × 1mm) and a sagittal 3D T2-weighted FLAIR (Fluid attenuation inversion recovery) (TR = 9000ms, TE = 93ms, TI = 2500ms, flip angle 120º, FOV = 256 × 200mm, voxel size = 1 × 1 × 1mm).

***Image analysis.*** 3D T1-weighted images were segmented with FreeSurfer (version 6.2) and the corresponding cortical thickness (CTh) and local gyrification index (lGI) of the parcellated regions was determined. Briefly, white matter points are chosen based on their locations in Talairach space as well as on their intensity and the local neighborhood intensities. Voxels were then classified as white matter or something other than white matter based on intensity and neighbor constraints. Cutting planes are chosen to separate the hemispheres from each other. An initial surface was then generated for each hemisphere by tiling the outside of the white matter mass for that hemisphere. This initial surface was then refined to follow the intensity gradients between the white and grey matter (this is referred to as the white surface). The white surface was then nudged to follow the intensity gradients between the gray matter and cerebrospinal fluid (this is the pial surface). The distance between the white and the pia gives us the thickness at each location of cortex [19, 20]. Once the PET images have been reconstructed, they were co-registered to the 3D T1-weighted image and the FreeSurfer parcellation was applied in order to obtain the mean value in each region. Brain PET data was normalized by the cerebellum white matter.

### Statistical analysis

 Descriptive and frequency statistics were obtained and comparisons made using the SPSS, version 21.0 for Windows. Nominal variables (aura, pain side and allodynia) were reported as frequencies (percentages) while mean ± standard deviation (age, headache frequency, migraine evolution, SF-36, HIT-6, MSQ, STAI, BDI-II, PSS and neuroimaging values) or median and interquartile range (IQR) (chronification, analgesic frequency and MIDAS) were reported for continuous variables. Normality assumption of quantitative variables was checked through visual methods (Q-Q plots) and normality tests (Shapiro-Wilk and Kolmogorov-Smirnov tests). The Levene test was used to test variance homogeneity.

Statistical significance between HC, EM and CM was assessed by Pearson’s chi-square when comparing categorical variables. In the case of having an expected count less than 5 in more than 20 % of cells in the contingency table, Fisher’s exact test was used. Clinical differences between EM and CM were assessed by independent t-test for continuous variables that followed a normal distribution (headache and analgesic frequencies, migraine evolution, HIT-6 and MSQ) and, Mann-Whitney U test was used for the rest of continuous variables (MIDAS). Pre-planned one-way ANOVA with Bonferroni correction in the post-hoc analysis was performed in order to find clinical differences between HC, EM and CM in the rest of continuous variables (Age, SF-36, STAI, BDI-II and PSS).

Metabolic (^18^ F-FDG-PET) and structural (CTh and lGI) values were analyzed using a pre-planned one-way ANCOVA, adjusted for the effect of age, for the study groups (EM, CM and HC). Following significant group main effects, post-hoc analysis was conducted using the Bonferroni correction in order to find differences in the neuroimaging variables (^18^ F-FDG-PET, CTh and lGI) between EM, CM and HC. Then, ^18^ F-FDG-PET measurements between groups were also analyzed adjusting by patient’s age and their CTh and lGI values in order to study if metabolic activity was influenced by brain structure. Finally, the degree of association between neuroimaging variables and clinical characteristics was computed by Pearson’s correlation coefficient and summarized by Pearson’s rho and related p-values.

A statistical power calculation was not previously conducted because this is an exploratory analysis and sample size was based on the available data. No missing data was obtained and p-values presented are for a two-tailed test and *p*-values < 0.05 were considered statistically significant.

## Results

### Demographical and clinical data

We included 17 right-handed women with migraine and 11 HC. After performing the neuroimaging protocol, we had to reject data from 3 subjects, as one showed abnormal ^18^ F-FDG-PET uptake (suggestive of another pathology), and 2 showed orthodontic appliance-derived artifacts that severely distorted the structural brain MRI. So, we finally included in the data analysis 15 migraine patients (8 EM and 7 CM) and 10 HC. The day of the scan, 3 CM patients reported mild headache (visual numerical scale 1–3).

Demographical and clinical characteristics of the sample are summarized in Tables 1 and 2. We did not find statistically significant differences regarding age, presence of aura, pain localization or migraine evolution. Mean headache frequency was 8.8 ± 1.8 days/month for the EM group and 24.7 ± 6.3 days/month for CM. CM patients showed higher scores in headache-disability MIDAS scale (EM: 9.5 [29.0] vs. CM: 32.0 [89.0], *p* = 0.039) although results from headache impact (HIT6) and quality of life (MSQ) were comparable between patients. We did no find differences regarding, stress, anxiety or depression scores between EM and CM patients. Statistically significant differences were found in BDI-II scores and SF-36 physical role, emotional role, bodily pain and health change domains between HC and CM.

**Table 1:** Demographic and clinical characteristics of patients and controls

	**HC** ***N = 10***	**EM** ***N = 8***	**CM** ***N = 7***	**HC-EM** **Adj.*P*value**	**HC-CM** **Adj.*P*value**	**EM-CM** **Adj.*P*value**
**Age**^a^, mean (SD), years	38.0 (14.3)	43.1 (12.2)	35.0 (17.0)	1.000	1.000	0.867
**STAI**^a^, mean (SD) * State* * Trait*	36.2 (7.8) 33.9 (6.5)	36.6 (5.1) 39.4 (6.4)	47.3 (12.8) 44.6 (12.1)	1.000 0.515	0.066 0.053	0.083 0.722
**BDI-II**^a^, mean (SD)	2.2 (2.2)	10.1 (8.6)	15.7 (11.1)	0.099	**0.003**	0.495
**SF-36**^a^, mean (SD) * Physical functioning* * Role physical* * Role emotional* * Vitality* * General mental health* * Social functioning* * Bodily pain* * General health* * Health change*	81.7 (25.9) 90.2 (38.9) 96.7 (10.0) 68.5 (18.2) 52.8 (31.2) 79.8 (21.0) 80.3 (23.4) 65.0 (21.0) 52.5 (17.5)	88.8 (9.5) 56.3 (54.7) 91.7 (15.4) 55.6 (19.4) 41.5 (19.4) 64.1 (26.3) 56.3 (20.1) 52.5 (16.5) 34.4 (18.6)	74.3 (16.7) 32.1 (23.8) 61.9 (40.5) 51.4 (12.8) 61.7 (16.8) 73.2 (19.7) 46.4 (17.3) 50.7 (24.2) 57.1 (12.2)	1.000 0.274 1.000 0.371 1.000 0.433 0.064 0.618 0.085	1.000 **0.024** **0.016** 0.160 1.000 1.000 **0.008** 0.499 1.000	0.514 0.818 0.064 1.000 0.383 1.000 1.000 1.000 **0.044**
**PSS**^a^, mean (SD)	14.5 (4.7)	20.1 (10.0)	22.0 (14.2)	0.675	0.369	1.000

Bold font in the *P* values column indicates statistically significant

^a^Significance assessed with one-way ANOVA with Bonferroni correction adjustment for multiple comparisons

*HC* healthy controls; *EM* episodic migraine; *CM* chronic migraine; *SD* standard deviation; *STAI* state-trait anxiety inventory; *BDI-II* Beck depression inventory II; *SF-36* 36-Item short form survey; *PSS* Perceived stress scale

**Table 2:** Migraine characteristics

	EM *N* = 8	CM *N* = 7	*P* value
**Headache frequency**^b^, mean (SD), days/month	8.8 (1.8)	24.7 (6.3)	**< 0.0001**
**Disease duration**^b^, mean (SD), years	28.4 (13.2)	20.0 (19.5)	0.343
**Chronification**, median (IQR), years	--	4.0 (11.0)	--
**Aura**^a^, n (%)	3 (37.5)	4 (57.1)	0.619
**Unilateral Pain**^a^, n (%)	5 (62.5)	7 (100.0)	0.200
**Analgesic Use**^c^, median (IQR), days/month	0.5 (8.8)	5.0 (8.0)	0.613
**MIDAS**^c^, median (IQR)	9.5 (29.0)	32.0 (89.0)	**0.029**
**HIT-6**^b^, mean (SD)	60.5 (3.9)	61.3 (4.9)	0.735
**MSQ**^b^, mean (SD) * Role Function preventive* * Role function restrictive* * Emotional function*	53.1 (23.8) 47.9 (17.1) 51.4 (27.2)	51.8 (15.8) 50.0 (13.3) 59.5 (16.3)	0.800 0.901 0.503

Bold font in the *P* values column indicates statistically significant

^a^Significance assessed with Fisher’s exact test

^b^Significance assessed with independent t-test

^c^Significance assessed with Mann-Whitney U test

*HC* healthy controls; *EM* episodic Migraine; *CM* chronic migraine; *SD* standard deviation; *IQR* interquartile range; *y* years; *d/mo* days per month; *MIDAS* migraine disability assessment; *HIT6* headache impact test 6; *MSQ* migraine-specific quality-of-life

### Image findings

#### Brain metabolic activity (^18^ F-FDG-PET findings)

**Fig. 1:**
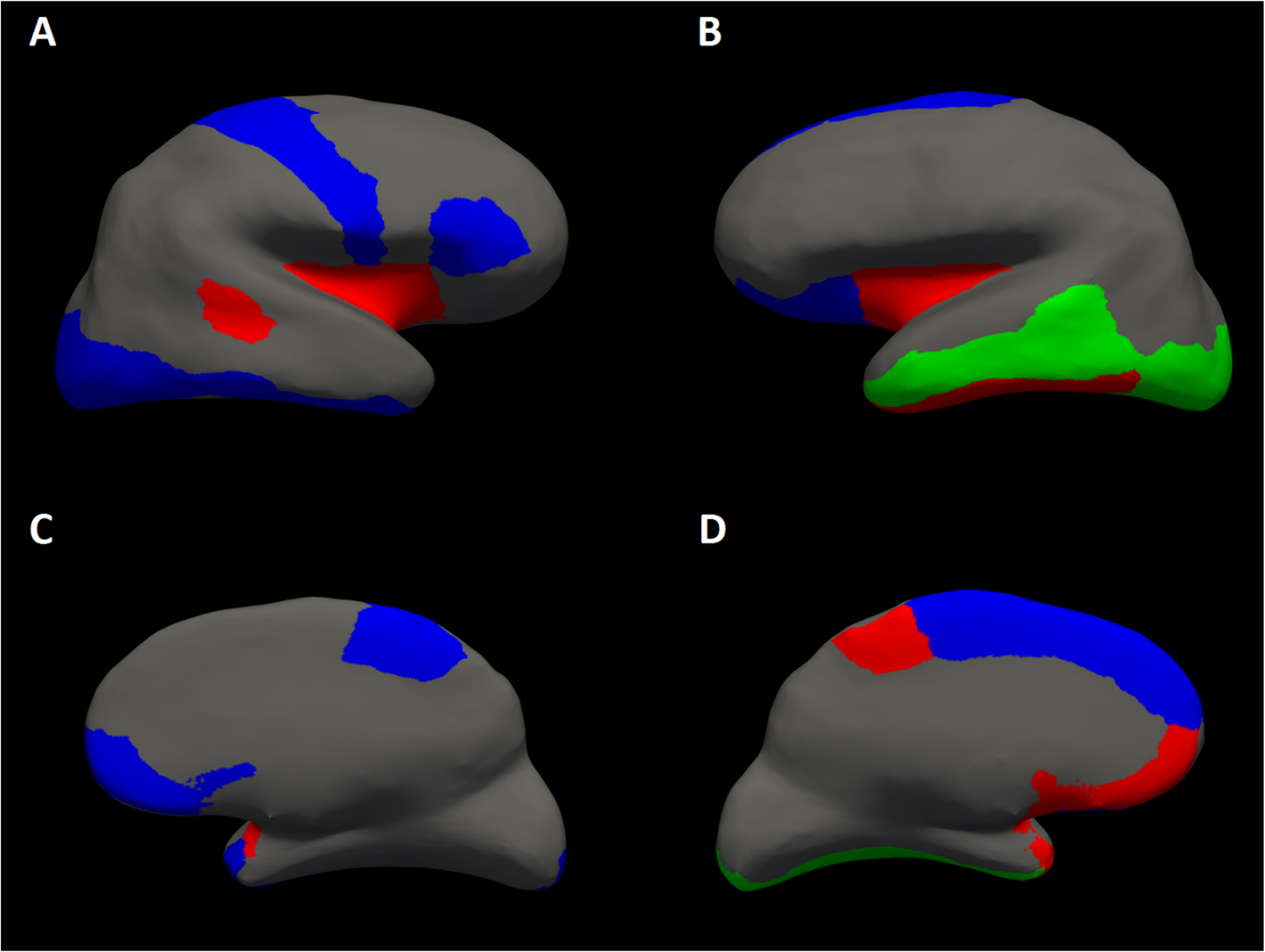
Statistically significant brain metabolic activity differences in ^18^FDG-PET (green), ^18^FDG-PET adjusted by brain structure (blue) or both analysis models (red) between healthy controls and chronic migraine. **A** Right hemisphere lateral view, (**B**) Left hemisphere lateral view, (**C**) Right hemisphere medial view, (**D**) Left hemisphere medial view

CM patients showed temporal hypometabolic areas compared with HC. Figure 1; Table 3 and Supplementary Table 1 show statistically significant group differences on 18 F-FDG-PET values, adjusted for patient’s age. CM patients showed a lower glucose metabolic activity compared to HC for a cluster of the left temporal lobe, which includes the left fusiform, left middle temporal, left inferior temporal, left temporal pole, bilateral insula and superior temporal sulcus. Frontal regions that showed differences between CM and HC were the left paracentral and the left medial orbitofrontal areas. Regarding the occipital lobe, only the left lateral occipital region showed an hypometabolism in CM versus HC. EM patients presented higher metabolic activity than CM but lower than HC for these regions (See Supplementary Table 1). However, only the metabolic activity for the bilateral temporal pole in EM showed statistically significant differences compared to HC. We did not find any differences in cerebral glucose metabolism when we compared both migraine subgroups together (CM and EM) with HC.

**Table 3:** Statistically significant brain metabolic activity (FDG-PET) differences adjusted by brain structure between healthy controls and migraine subgroups (CM and EM)

Anatomical Areas, mean (SD)	HC (*N *= 10)	EM (*N* = 8)	CM (*N *= 7)	Adj. *P* Value			HC (*N* = 10)	EM (*N* = 8)	CM (*N* = 7)	Adj. *P* Value		
				**HC-EM**	**HC-CM**	**EM-CM**				**HC-EM**	**HC-CM**	**EM-CM**
	**LEFT side**						**RIGHT side**					
Frontal Areas												
Lateral Orbitofrontal	1.605 (0.029)	1.552 (0.036)	1.503 (0.036)	0.296	**0.038**	0.366	1.598 (0.033)	1.527 (0.040)	1.492 (0.041)	0.201	0.057	0.562
Medial Orbitofrontal	1.564 (0.034)	1.522 (0.043)	1.413 (0.047)	0.453	**0.018**	0.132	1.590 (0.033)	1.493 (0.038)	1.469 (0.041)	0.067	**0.033**	0.686
Paracentral	1.443 (0.033)	1.438 (0.040)	1.309 (0.042)	0.919	**0.019**	**0.042**	1.473 (0.034)	1.438 (0.038)	1.345 (0.041)	0.503	**0.030**	0.116
Pars Triangularis	1.724 (0.033)	1.689 (0.040)	1.626 (0.043)	0.506	0.085	0.313	1.749 (0.036)	1.644 (0.041)	1.625 (0.044)	0.074	**0.043**	0.763
Precentral	1.559 (0.033)	1.538 (0.040)	1.462 (0.043)	0.703	0.095	0.217	1.560 (0.030)	1.550 (0.035)	1.446 (0.037)	0.821	**0.029**	0.070
Superior Frontal	1.623 (0.037)	1.591 (0.043)	1.490 (0.050)	0.572	**0.050**	0.187	1.607 (0.034)	1.591 (0.040)	1.504 (0.046)	0.766	0.090	0.201
Frontal Pole	1.702 (0.040)	1.645 (0.049)	1.602 (0.051)	0.385	0.135	0.551	1.778 (0.042)	1.696 (0.047)	1.632 (0.050)	0.220	**0.039**	0.354
Temporal areas												
Banks Superior Temporal Sulcus	1.548 (0.034)	1.470 (0.040)	1.444 (0.044)	0.152	0.083	0.673	1.584 (0.032)	1.507 (0.035)	1.459 (0.038)	0.127	**0.026**	0.372
Inferior Temporal	1.449 (0.026)	1.392 (0.031)	1.354 (0.034)	0.183	**0.039**	0.426	1.447 (0.023)	1.382 (0.026)	1.362 (0.029)	0.076	**0.035**	0.631
Temporal Pole	1.137 (0.021)	1.045 (0.024)	1.038 (0.026)	**0.011**	**0.008**	0.840	1.098 (0.021)	0.994 (0.025)	1.005 (0.025)	**0.007**	**0.031**	0.357
Insula	1.373 (0.027)	1.311 (0.032)	1.281 (0.035)	0.162	**0.050**	0.541	1.388 (0.028)	1.309 (0.032)	1.287 (0.033)	0.080	**0.031**	0.649
Occipital Areas												
Lateral Occipital	1.550 (0.036)	1.536 (0.042)	1.436 (0.045)	0.815	0.069	0.122	1.630 (0.044)	1.582 (0.048)	1.476 (0.051)	0.489	**0.036**	0.143

Bold font in the *P* values column indicates statistically significant

Significance assessed with ANCOVA modelling adjusted by patients’ age and brain structural variables (cortical thickness and gyrification index) in order to find metabolic activity differences between healthy controls and migraine patients (EM and CM)

*HC* healthy controls; *EM* episodic Migraine; *CM* chronic migraine *SD* standard deviation; *Adj. P value adjusted* *P* value (Bonferroni corrected)

#### Cortical Thickness (CTh)

CTh age-adjusted analysis showed frontal CTh increase in CM compared with HC (See Table 4 and Supplementary Table 2): bilateral caudal middle frontal, left medial orbitofrontal, bilateral rostral middle frontal and left superior frontal. We also found a statistically significant higher CTh in EM respect to HC in the right parahippocampal region and right inferior parietal region. EM patients presented intermediate CTh values between CM and HC for the previous areas; only CTh values for right pars opercularis, right transverse temporal and right temporal pole were statistically significant thinner in EM in comparison to HC. Finally, between EM and CM, we found that left rostral middle frontal, left caudal middle frontal and right superior frontal showed a statistically significant thinning in EM compared to CM.

**Table 4:** Statistically significant cortical thickness differences between healthy controls and migraine subgroups (CM and EM)

Anatomical Areas, mean (SD)	HC (*N* = 10)	EM (*N* = 8)	CM (*N* = 7)	Adj. *P* Value			HC (*N* = 10)		EM (*N* = 8)	CM (*N* = 7)	Adj. *P* Value		
				**HC-EM**	**HC-CM**	**EM-CM**					**HC-EM**	**HC-CM**	**EM-CM**
	**LEFT side**						**RIGHT side**						
Frontal Areas													
Caudal Middle Frontal	2.480 (0.054)	2.481 (0.063)	2.608 (0.069)	0.986	**0.028**	**0.043**	2.414 (0.067)		2.473 (0.080)	2.578 (0.080)	0.389	**0.040**	0.138
Medial Orbitofrontal	2.317 (0.067)	2.378 (0.057)	2.472 (0.072)	0.377	**0.025**	0.093	2.330 (0.049)		2.305 (0.059)	2.367 (0.062)	0.748	0.642	0.480
Pars Opercularis	2.540 (0.040)	2.596 (0.048)	2.609 (0.041)	0.386	0.302	0.857	2.638 (0.039)		2.469 (0.047)	2.604 (0.050)	**0.011**	0.587	0.064
Rostral Middle Frontal	2.305 (0.043)	2.287 (0.050)	2.415 (0.054)	0.688	**0.022**	**0.016**	2.186 (0.044)		2.217 (0.054)	2.316 (0.058)	0.508	**0.022**	0.052
Superior Frontal	2.673 (0.052)	2.684 (0.062)	2.793 (0.067)	0.836	**0.028**	0.062	2.630 (0.031)		2.577 (0.037)	2.707 (0.039)	0.286	0.136	**0.026**
Temporal areas													
Transverse Temporal	2.451 (0.067)	2.291 (0.080)	2.469 (0.085)	0.141	0.868	0.146	2.506 (0.058)		2.318 (0.069)	2.435 (0.073)	**0.049**	0.460	0.260
Parahippocampal	2.892 (0.087)	2.776 (0.104)	2.693 (0.110)	0.405	0.172	0.595	2.944 (0.100)		2.777 (0.115)	2.705 (0.122)	0.080	**0.018**	0.488
Temporal Pole	3.717 (0.082)	3.620 (0.098)	3.720 (0.104)	0.459	0.983	0.498	4.021 (0.086)		3.677 (0.103)	3.997 (0.100)	**0.018**	0.863	0.057
Parietal Areas													
Inferior Parietal	2.414 (0.034)	2.441 (0.040)	2.478 (0.043)	0.625	0.252	0.530	2.452 (0.050)		2.465 (0.060)	2.559 (0.052)	0.787	**0.048**	0.108

Bold font in the *P* values column indicates statistically significant

Significance assessed with ANCOVA modelling adjusted by patients’ age in order to find cortical thickness differences between healthy controls and migraine patients (EM and CM)

*HC* healthy controls; *EM* episodic migraine; *CM* chronic migraine; *SD* standard deviation; *Adj. P value* adjusted *P* value (Bonferroni correction)

#### Local Gyrification Indexes (lGI)

No statistically significant differences (See Supplementary Table 3) were found in the local gyrification ratings (adjusted by patient’s age) between study groups (HC vs. EM vs. CM).

#### Cerebral metabolic activity adjusted by brain structure

We studied the influence of brain structure in the metabolic activity. Hence, we analyzed again brain metabolic activity adjusted by age, CTh and lGI (See Table 3). In comparison with HC, we found that CM presented and hypometabolism in several frontal regions: left lateral orbitofrontal, bilateral medial orbitofrontal, bilateral paracentral, left superior frontal and right frontal pole. For the temporal lobe, statistically significant differences were also found in right superior temporal sulcus, bilateral inferior temporal, bilateral temporal pole and bilateral insula between HC and CM. The right lateral occipital presented also statistically significant lower metabolism in CM. Regarding to EM, we found a statistically significant hypometabolism in comparison to HC in the bilateral temporal pole.

#### Clinical correlation with imaging measurements

In order to further investigate the association between metabolism and structural measurements with clinical variables, we performed the following correlation analysis. Regarding metabolism activity, we observed that a longer migraine duration was correlated with hypometabolism of the left lateral orbitofrontal (L: r= -0.572, *p* = 0.026), bilateral medial orbitofrontal (L: r= -0.642, *p* = 0.010 and R: r= -0.616, *p* = 0.014), left inferior temporal (L: r= -0.518, *p* = 0.048), bilateral temporal pole (L: r= -0.558, *p* = 0.031 and R: r= -0.516, *p* = 0.049), bilateral insula (L: r= -0.656, *p *= 0.008 and R: r= -0.716, *p* = 0.003) and bilateral lateral occipital (L: r= -0.414, *p* = 0.036 and R: r= -0.391, *p* = 0.048). We also found a negative correlation between headache frequency and brain metabolism in the left paracentral region (L: r= -0.417, *p* = 0.034). The left banks of superior temporal sulcus (L: r=-0.638, *p* = 0.011), left middle temporal (L: r=-0.570, *p* = 0.027) and left inferior temporal (L: r=-0.589, *p* = 0.021) metabolism were negatively correlated to analgesic intake. In regards to CTh, a positive correlation was found with disease duration and left medial orbitofrontal (L: r = 0.405, *p* = 0.040), left rostral middle frontal (L: r = 0.424, *p* = 0.031), left superior frontal (L: r = 0.443, *p* = 0.023) and right inferior parietal (R: r = 0.443, *p* = 0.024). A statistically significant negative correlation was also found between medial orbitofrontal area and headache frequency (L: r= -0.554, *p* = 0.036). We did not find any statistically significant correlation with lGI and clinical variables.

## Discussion

This is the first study which explores metabolic and structural differences in migraine patients combining PET and MRI techniques. Our results suggest that CM patients show cortical frontotemporal structural and metabolic differences when compared to HC. It is important to point out that we have included structural data to adjust cortical metabolism analysis, and we have found that the structural adjusted ^18^ F-FDG-PET values may increase the sensitivity of the model, showing more areas with statistical differences than the non-adjusted model.

Our data show that CM patients have a decreased metabolism in prefrontal areas including frontal pole, superior and inferior frontal cortex as well as orbitofrontal areas. Prefrontal structures have been related to cognitive, affective and sensory processing functions. Previous imaging studies have demonstrated that the prefrontal cortex, connected to limbic regions, can regulate the perception and behavioral expression of pain in humans [21]. So, frontal-limbic dysfunction could be involved in chronic pain processing, as well as, in chronic migraine. On the other hand, persistent orbitofrontal cortex hypometabolism has been found in CM with medication overuse patients after withdrawal [22], as well as, with other substance addictions [23] so, it has been suggested that this structure may be linked to the dependence drive [24, 25]. However, this finding has been more recently replicated also in a group of non-medication-overuse migraineurs compared to HC [26], suggesting that this finding is genuinely associated with migraine rather than medication overuse. Our group of participants was thoroughly screened for current or past history of medication overuse which makes us think that this finding would be linked to affective aspects or migraine but not exclusively with medication use. Apart from prefrontal areas, involved in executive and behaviour control functions, we have also found hypometabolism of paracentral and precentral structures, that are likely to mediate part of the cognitive dimension of pain-processing associated with localization and ‘motor’ response to pain [21].

Besides frontal hypometabolism, our results suggest that CM patients have a hypometabolism in the bilateral inferior temporal lobe, temporal pole and bilateral insula compared to HC. Previous studies also found a temporal dysfunction in patients with EM during the interictal period. Kim et al. [27] found that migraine patients had significant hypometabolism in the bilateral insula and superior temporal cortex. Although this study does not include CM patients specifically, they found a significant negative correlation with estimated lifetime headache frequency and right insula metabolism, which could indirectly point to chronification. Insular hypometabolism was also found in a group of patients with CM and medication overuse. However, metabolism of this area normalized after analgesic withdrawal showing no statistical differences with HC [22]. Similar to our study, MRI studies in EM and CM patients have also demonstrated enhanced stimulus-induced activation and structural differences of the temporal pole [25–27], a region that integrates auditory, olfactory, visual, and painful stimuli.

There are few brain PET-imaging reports on chronic nociceptive pain other than headache disorders. A number of studies have investigated the cerebral response pattern to painful, usually allodynic, stimulation in chronic neuropathic pain models showing metabolic changes in multiple brain areas including orbitofrontal cortex, somatosensory cortex, anterior cingulate cortex, anterior insula, thalamus brainstem, and cerebellum [28] which have been identified as pain processing areas.

Cortical metabolism values in EM patients were in between HC and CM for all areas, except for right temporal pole. EM showed statistically significant bilateral temporal pole hypometabolism compared to HC and an increased metabolism for the left paracentral compared to CM. All other comparations did not reach statistical significance. This might point to a relationship between migraine chronification and decreased cortical metabolism.

In relation to structural data, to date, the number of morphometric studies that include CM patients has been scarce and has not been able to establish which areas could be involved in the chronification process. Only two previously published studies have explored differences in CTh of CM patients compared to HC. One showed significantly thinner cortices in the bilateral insular cortex, caudal middle frontal gyrus, precentral gyrus, and parietal lobes in patients with CM [28], while the other has not found significant differences between CM patients and controls [29]. Due to the inconsistency of previous morphometric data on CM and the small number of patients included in our study, we need to be cautious when interpreting our structural data.

In regards to the correlation between clinical characteristics and imaging measurements, we found that temporal areas metabolism, including insula and orbitofrontal cortex, was correlated with migraine disease duration. We also found negative correlations between metabolism values in temporal areas and analgesic use as well as metabolism in left paracentral area and headache frequency. Previous PET studies have not explored clinical correlations. MRI studies performed in CM patients have found some correlations between migraine duration, headache frequency as well as other clinical variables and temporal sulcus, insula CTh [29, 30] or cerebellum grey matter volumes [31].

Finally, a point that needs further discussion is the joint analysis of structural and metabolic data. Since it has been reported that migraine patients show regional cortical volume and thickness changes [32], we can expect that the structural changes may cause differences in PET intensity due to anatomy rather than activity itself or *viceversa*, blurring the differences between groups. To our knowledge, no surface-based analyses using precise structural anatomical information have been applied to the statistical analysis of PET images in migraine research. Our ^18^FDG-PET data has been analysed adjusting by patient’s age and their CTh and lGI values in order to minimize the influence of cortical structure on metabolic activity. In the frontal lobe, where we have found remarkable structural differences between CM and HC, a structural-adjusted approach has increased the number of frontal areas that showed differences from 2 to 9. In contrast, this method confirms only 5 over 9 temporal areas, specifically the right temporal pole and inferior temporal. As scientific data available up to now suggest that structural changes may underlie migraine pathophysiology, we believe that structural data should be always taken into consideration when metabolic imaging studies are performed. 

Our study has several strengths. This is the first study that explores metabolic and structural differences in migraine patients combining PET and MRI techniques, that were done on the same day, avoiding any possible biases in relation to natural migraine fluctuations. ^18^ F-FDG uptake values have been adjusted by structural measurements in each corresponding region, providing a more accurate measure of cortical metabolic activity. Secondly, the selection of participants was strictly and meticulously performed and HC were also recruited specifically for this study and were also screened for headache family history or recurrent headaches. These strict inclusion and exclusion criteria increase the internal validity of our findings and allow us to attribute the differences found to CM and not to other confounders. The main limitation is the low number of patients included that forces us to be cautious in data interpretation. Finally, although patients where scanned outside of a migraine attack, three patients had mild pain and, we cannot exclude interferences with mild pain, premonitory or postdromal changes in brain metabolism. In spite of the limitations, we believe that our findings are a reliable basis to develop future projects and increase our understanding on migraine pathophysiology.

## Conclusions

CM is correlated with brain metabolic and structural differences. CM patients showed frontotemporal hypometabolism and increased frontal cortical thickness when compared to HC. EM patients presented higher metabolic activity than CM but lower than HC for these regions which follows a headache frequency-related spectrum of change. Metabolic data analysis using precise structural anatomical information allowed us to obtain more accurate models. So, combined information from sequential or simultaneous PET and MRI could optimize the study of functional neurological disorders such as migraine.

## Supplementary information

**Additional file 1.**

## Data Availability

The datasets used and/or analysed during the current study are available from the corresponding author on reasonable request.

## Abbreviations

MRI: Magnetic Resonance Imaging
CM: Chronic Migraine
^18^F-FDG: ^18^F-Fluorodeoxyglucose Positron Emission Tomography
BDI-II: Beck Depression Inventory II
CT: Computed Tomography
CTh: Cortical Thickness
EM: Episodic Migraine
HC: Healthy Controls
HIT-6: Headache Impact Test 6
IGI: Local Gyrification Index
IQR: Interquartile Range
MIDAS: Migraine Disability Assessment
MSQ: Migraine-Specific Quality of Life Questionnaire
PSS: Perceived Stress Scale
SF-36: 36-Items Short Form Health Survey
STAI: State-Trait Anxiety Inventory

## References

[1] Lipton RB (2009). Tracing transformation: chronic migraine classification, progression, and epidemiology. Neurology.

[2] Torres-Ferrús M, Ursitti F, Alpuente-Ruiz A, Brunello F, Chiappino D (2020). From transformation to chronification of migraine: pathophysiological and clinical aspects. J Headache Pain J Headache Pain.

[3] Pozo-Rosich P, Coppola G, Pascual J, Schwedt TJ. How does the brain change in chronic migraine? Developing disease biomarkers. Cephalalgia. 2020;333102420974359 10.1177/0333102420974359 33291995

[4] Neeb L, Bastian K, Villringer K, Israel H, Reuter U, Fiebach JB. Structural Gray Matter Alterations in Chronic Migraine: Implications for a Progressive Disease? Headache. 2017;57(3):400–16 10.1111/head.13012 28028808

[5] Chen Z, Chen X, Liu M, Liu S, Ma L, Yu S (2017). Volume expansion of periaqueductal gray in episodic migraine: a pilot MRI structural imaging study. J Headache Pain.

[6] Liu H-Y, Chou K-H, Lee P-L, Fuh J-L, Niddam DM, Lai K-L (2017). Hippocampus and amygdala volume in relation to migraine frequency and prognosis. Cephalalgia.

[7] Dominguez C, Lopez A, Ramos-Cabrer P, Vieites-Prado A, Perez-Mato M, Villalba C (2019). Iron deposition in periaqueductal gray matter as a potential biomarker for chronic migraine. Neurology.

[8] Schulte LH, Allers A, May A (2017). Hypothalamus as a mediator of chronic migraine: Evidence from high-resolution fMRI. Neurology.

[9] Schöll M, Damián A, Engler H (2014). Fluorodeoxyglucose PET in Neurology and Psychiatry. PET Clin.

[10] Schulte LH, May A (2016). Functional Neuroimaging in Migraine: Chances and Challenges. Headache.

[11] Headache Classification Committee of the International Headache Society (IHS). The International Classification of Headache Disorders, 3rd edition (beta version). Cephalalgia. 2013;33(9):629–808 10.1177/0333102413485658 23771276

[12] Spielberger CD, Gorsuch RL, Lushene R, Vagg PR, Jacobs GA (1983). Manual for the State-Trait Anxiety Inventory.

[13] Beck AT, Steer RA, Brown GK (1996). Manual for the Beck Depression Inventory-II.

[14] Cohen S, Kamarck T, Mermelstein R (1983). A Global Measure of Perceived Stress. J Health Soc Behav.

[15] Ware JEJ, Sherbourne CD (1992). The MOS 36-item short-form health survey (SF-36). I. Conceptual framework and item selection. Med Care.

[16] Stewart WF, Lipton RB, Dowson AJ, Sawyer J (2001) Development and testing of the Migraine Disability Assessment (MIDAS) Questionnaire to assess headache-related disability. Neurology. Mar 1;56(suppl 1):S20 LP-S28 10.1212/wnl.56.suppl_1.s20 11294956

[17] Kosinski M, Bayliss MS, Bjorner JB, Ware JEJ, Garber WH, Batenhorst A (2003). A six-item short-form survey for measuring headache impact: the HIT-6. Qual Life Res.

[18] Martin BC, Pathak DS, Sharfman MI, Adelman JU, Taylor F, Kwong WJ (2000). Validity and reliability of the migraine-specific quality of life questionnaire (MSQ Version 2.1). Headache.

[19] Dale AM, Fischl B, Sereno MI (1999). Cortical surface-based analysis. I. Segmentation and surface reconstruction. Neuroimage.

[20] Fischl B, Dale AM (2000). Measuring the thickness of the human cerebral cortex from magnetic resonance images. Proc Natl Acad Sci U S A.

[21] Lee MC, Tracey I, Colvin L, Rowbotham DJ (2013). Imaging pain: A potent means for investigating pain mechanisms in patients. Br J Anaesth.

[22] Fumal A, Laureys S, Di Clemente L, Boly M, Bohotin V, Vandenheede M (2006). Orbitofrontal cortex involvement in chronic analgesic-overuse headache evolving from episodic migraine. Brain.

[23] Volkow ND, Fowler JS (2000). Addiction, a disease of compulsion and drive: involvement of the orbitofrontal cortex. Cereb Cortex.

[24] Fumal A, Laureys S, Di Clemente L, Boly M, Bohotin V, Vandenheede M (2006). Orbitofrontal cortex involvement in chronic analgesic-overuse headache evolving from episodic migraine. Brain.

[25] Riederer F, Gantenbein AR, Marti M, Luechinger R, Kollias S, Sándor PS (2013). Decrease of Gray Matter Volume in the Midbrain is Associated with Treatment Response in Medication-Overuse Headache: Possible Influence of Orbitofrontal Cortex. J Neurosci.

[26] Magis D, D’Ostilio K, Thibaut A, De Pasqua V, Gerard P, Hustinx R (2017). Cerebral metabolism before and after external trigeminal nerve stimulation in episodic migraine. Cephalalgia.

[27] Kim JH, Kim S, Suh S-I, Koh S-B, Park K-W, Oh K (2010). Interictal metabolic changes in episodic migraine: a voxel-based FDG-PET study. Cephalalgia.

[28] Kupers R, Kehlet H (2006). Brain imaging of clinical pain states: a critical review and strategies for future studies. Lancet Neurol.

[29] Lai K-L, Niddam DM, Fuh J-L, Chen W-T, Wu J-C, Wang S-J (2016). Cortical morphological changes in chronic migraine in a Taiwanese cohort: Surface- and voxel-based analyses. Cephalalgia.

[30] Woldeamanuel YW, DeSouza DD, Sanjanwala BM, Cowan RP (2019). Clinical Features Contributing to Cortical Thickness Changes in Chronic Migraine - A Pilot Study. Headache.

[31] Coppola G, Petolicchio B, Di Renzo A, Tinelli E, Lorenzo C, Di, Parisi V et al. Cerebral gray matter volume in patients with chronic migraine: correlations with clinical features The Journal of Headache and Pain. J Headache Pain. 2017;18 10.1186/s10194-017-0825-z PMC5762618 29322264

[32] Filippi M, Messina R (2019). The Chronic Migraine Brain: What Have We Learned From Neuroimaging?. Front Neurol.

