# Supplementary material for: Cortical metabolic and structural differences in patients with chronic migraine. An exploratory 18FDG-PET and MRI study
Source: J Headache Pain. 2021 Jul 17;22(1):75. doi: 10.1186/s10194-021-01289-5 (PMC8285838; doi:10.1186/s10194-021-01289-5)
Supplement: Supplementary file 1 — Additional file 1. [file 10194_2021_1289_MOESM1_ESM.docx]

| **SUPPLEMENTARY TABLE 1. Brain metabolic activity (FDG-PET) differences between healthy controls and migraine subgroups (CM and EM)** | | | | | | | | |  | | | |
| --- | --- | --- | --- | --- | --- | --- | --- | --- | --- | --- | --- | --- |
| **Anatomical Areas**, mean (SD) | **HC**  **(N = 10)** | **EM**  **(N = 8)** | **CM**  **(N = 7)** | **Adj. *P* Value** | | | **HC**  **(N = 10)** | **EM**  **(N = 8)** | **CM**  **(N = 7)** | **Adj. *P* Value** | | |
|  |  |  |  | **HC-EM** | **HC-CM** | **EM-CM** |  |  |  | **HC-EM** | **HC-CM** | **EM-CM** |
|  | **LEFT side** | | | | | | **RIGHT side** | | | | | |
| Frontal Areas |  |  |  |  |  |  |  |  |  |  |  |  |
| Caudal Anterior Cingulate | 1.495 (0.030) | 1.428 (0.036) | 1.415 (0.038) | 0.164 | 0.111 | 0.810 | 1.514 (0.040) | 1.423 (0.047) | 1.423 (0.050) | 0.158 | 0.169 | 0.998 |
| Caudal Middle Frontal | 1.740 (0.037) | 1.711 (0.044) | 1.649 (0.047) | 0.628 | 0.142 | 0.349 | 1.698 (0.035) | 1.695 (0.042) | 1.665 (0.045) | 0.956 | 0.567 | 0.634 |
| Lateral Orbitofrontal | 1.603 (0.031) | 1.545 (0.037) | 1.514 (0.039) | 0.250 | 0.085 | 0.568 | 1.595 (0.035) | 1.499 (0.041) | 1.516 (0.044) | 0.091 | 0.170 | 0.791 |
| **Medial Orbitofrontal** | 1.563 (0.044) | 1.493 (0.050) | 1.447 (0.053) | 0.208 | **0.050** | 0.462 | 1.573 (0.033) | 1.490 (0.039) | 1.482 (0.041) | 0.116 | 0.098 | 0.899 |
| **Paracentral** | 1.449 (0.041) | 1.420 (0.048) | 1.320 (0.049) | 0.589 | **0.028** | 0.109 | 1.459 (0.032) | 1.431 (0.038) | 1.362 (0.041) | 0.584 | 0.076 | 0.239 |
| Pars Opercularis | 1.759 (0.037) | 1.702 (0.044) | 1.641 (0.047) | 0.335 | 0.058 | 0.351 | 1.757 (0.038) | 1.704 (0.045) | 1.679 (0.048) | 0.377 | 0.212 | 0.710 |
| Pars Orbitalis | 1.757 (0.040) | 1.707 (0.048) | 1.673 (0.051) | 0.434 | 0.206 | 0.632 | 1.749 (0.040) | 1.673 (0.047) | 1.669 (0.050) | 0.230 | 0.220 | 0.953 |
| Pars Triangularis | 1.723 (0.032) | 1.691 (0.038) | 1.624 (0.040) | 0.532 | 0.067 | 0.243 | 1.728 (0.035) | 1.650 (0.042) | 1.635 (0.045) | 0.170 | 0.115 | 0.810 |
| Precentral | 1.568 (0.034) | 1.531 (0.041) | 1.456 (0.043) | 0.493 | 0.053 | 0.224 | 1.552 (0.032) | 1.524 (0.038) | 1.480 (0.041) | 0.581 | 0.176 | 0.443 |
| Rostral Anterior Cingulate | 1.489 (0.036) | 1.441 (0.043) | 1.393 (0.046) | 0.407 | 0.113 | 0.455 | 1.415 (0.039) | 1.338 (0.046) | 1.357 (0.049) | 0.212 | 0.359 | 0.779 |
| Rostral Middle Frontal | 1.737 (0.035) | 1.705 (0.042) | 1.643 (0.045) | 0.567 | 0.114 | 0.333 | 1.719 (0.037) | 1.657 (0.044) | 1.647 (0.047) | 0.302 | 0.245 | 0.878 |
| Superior Frontal | 1.614 (0.035) | 1.587 (0.042) | 1.517 (0.045) | 0.629 | 0.104 | 0.276 | 1.591 (0.034) | 1.569 (0.040) | 1.545 (0.042) | 0.684 | 0.409 | 0.690 |
| Frontal Pole | 1.696 (0.039) | 1.655 (0.046) | 1.600 (0.049) | 0.505 | 0.137 | 0.425 | 1.744 (0.040) | 1.710 (0.048) | 1.648 (0.051) | 0.597 | 0.154 | 0.391 |
| Temporal areas |  |  |  |  |  |  |  |  |  |  |  |  |
| **Banks Superior Temporal Sulcus** | 1.551 (0.042) | 1.480 (0.050) | 1.429 (0.053) | 0.200 | **0.039** | 0.412 | 1.575 (0.038) | 1.504 (0.044) | 1.467 (0.046) | 0.141 | **0.035** | 0.487 |
| Entorhinal | 1.085 (0.019) | 1.051 (0.023) | 1.064 (0.024) | 0.264 | 0.508 | 0.693 | 1.081 (0.021) | 1.053 (0.025) | 1.042 (0.026) | 0.397 | 0.260 | 0.773 |
| **Fusiform** | 1.388 (0.027) | 1.336 (0.031) | 1.312 (0.033) | 0.163 | **0.050** | 0.556 | 1.398 (0.026) | 1.347 (0.031) | 1.318 (0.033) | 0.233 | 0.075 | 0.540 |
| Superior Temporal | 1.346 (0.023) | 1.296 (0.028) | 1.270 (0.030) | 0.185 | 0.058 | 0.547 | 1.362 (0.025) | 1.309 (0.030) | 1.290 (0.032) | 0.190 | 0.091 | 0.681 |
| **Middle Temporal** | 1.528 (0.033) | 1.473 (0.039) | 1.430 (0.041) | 0.201 | **0.032** | 0.366 | 1.544 (0.027) | 1.484 (0.032) | 1.478 (0.034) | 0.172 | 0.146 | 0.901 |
| **Inferior Temporal** | 1.452 (0.030) | 1.387 (0.035) | 1.354 (0.036) | 0.109 | **0.023** | 0.454 | 1.438 (0.023) | 1.382 (0.028) | 1.363 (0.030) | 0.142 | 0.059 | 0.643 |
| Transverse Temporal | 1.928 (0.046) | 1.852 (0.055) | 1.821 (0.059) | 0.301 | 0.163 | 0.707 | 1.946 (0.044) | 1.887 (0.052) | 1.850 (0.055) | 0.394 | 0.188 | 0.643 |
| Isthmus of cingulate | 1.712 (0.041) | 1.720 (0.048) | 1.614 (0.052) | 0.893 | 0.152 | 0.154 | 1.726 (0.043) | 1.689 (0.051) | 1.641 (0.054) | 0.585 | 0.231 | 0.531 |
| Parahippocampal | 1.191 (0.021) | 1.142 (0.025) | 1.148 (0.026) | 0.143 | 0.200 | 0.890 | 1.194 (0.020) | 1.141 (0.023) | 1.148 (0.025) | 0.093 | 0.153 | 0.844 |
| **Temporal Pole** | 1.130 (0.022) | 1.051 (0.026) | 1.042 (0.027) | **0.023** | **0.015** | 0.813 | 1.084 (0.022) | 1.005 (0.026) | 1.043 (0.028) | **0.028** | 0.245 | 0.318 |
| **Insula** | 1.375 (0.033) | 1.313 (0.038) | 1.276 (0.040) | 0.162 | **0.037** | 0.460 | 1.381 (0.031) | 1.304 (0.035) | 1.290 (0.037) | 0.071 | **0.050** | 0.862 |
| Parietal Areas |  |  |  |  |  |  |  |  |  |  |  |  |
| Superior Parietal | 1.420 (0.028) | 1.449 (0.033) | 1.365 (0.035) | 0.504 | 0.235 | 0.100 | 1.433 (0.029) | 1.457 (0.034) | 1.403 (0.036) | 0.596 | 0.526 | 0.297 |
| Inferior Parietal | 1.543 (0.034) | 1.563 (0.041) | 1.468 (0.043) | 0.703 | 0.188 | 0.127 | 1.589 (0.032) | 1.591 (0.038) | 1.537 (0.040) | 0.974 | 0.314 | 0.342 |
|  |  |  |  |  |  |  |  |  |  |  |  |  |
| Postcentral | 1.415 (0.030) | 1.415 (0.036) | 1.320 (0.038) | 0.989 | 0.064 | 0.090 | 1.405 (0.032) | 1.426 (0.038) | 1.367 (0.041) | 0.680 | 0.473 | 0.310 |
| Posterior Cingulate | 1.745 (0.042) | 1.712 (0.050) | 1.684 (0.053) | 0.623 | 0.377 | 0.706 | 1.741 (0.041) | 1.680 (0.049) | 1.687 (0.052) | 0.351 | 0.418 | 0.929 |
| Precuneus | 1.728 (0.032) | 1.707 (0.038) | 1.627 (0.040) | 0.680 | 0.059 | 0.162 | 1.715 (0.030) | 1.686 (0.035) | 1.639 (0.037) | 0.523 | 0.128 | 0.384 |
|  |  |  |  |  |  |  |  |  |  |  |  |  |
| Supramarginal | 1.502 (0.035) | 1.511 (0.041) | 1.437 (0.044) | 0.874 | 0.251 | 0.236 | 1.497 (0.032) | 1.494 (0.038) | 1.465 (0.040) | 0.950 | 0.534 | 0.607 |
| Occipital Areas |  |  |  |  |  |  |  |  |  |  |  |  |
| Cuneus | 1.657 (0.036) | 1.681 (0.043) | 1.628 (0.046) | 0.678 | 0.625 | 0.417 | 1.694 (0.045) | 1.692 (0.053) | 1.652 (0.056) | 0.981 | 0.565 | 0.613 |
| **Lateral Occipital** | 1.558 (0.042) | 1.530 (0.051) | 1.429 (0.051) | 0.607 | **0.029** | 0.109 | 1.608 (0.040) | 1.583 (0.047) | 1.484 (0.050) | 0.688 | 0.066 | 0.173 |
| Lingual | 1.633 (0.032) | 1.622 (0.038) | 1.545 (0.040) | 0.816 | 0.098 | 0.184 | 1.643 (0.031) | 1.630 (0.037) | 1.573 (0.040) | 0.787 | 0.179 | 0.315 |
| Pericalcarine | 1.895 (0.059) | 1.854 (0.071) | 1.796 (0.075) | 0.663 | 0.313 | 0.585 | 1.968 (0.067) | 1.935 (0.080) | 1.859 (0.085) | 0.759 | 0.326 | 0.526 |

Bold font in the *P* values column indicates statistically significant.

Significance assessed with ANCOVA modelling adjusted by patients’ age in order to find FDG-PET differences between healthy controls and migraine patients (EM and CM).

HC: healthy controls, EM: episodic migraine, CM: chronic migraine, SD: standard deviation; Adj. *P* value: adjusted *P* value (Bonferroni correction)

| **SUPPLEMENTARY TABLE 2. Cortical thickness differences between healthy controls and migraine subgroups (CM and EM)** | | | | | | | | |  | | | |
| --- | --- | --- | --- | --- | --- | --- | --- | --- | --- | --- | --- | --- |
| **Anatomical Areas**, mean (SD) | **HC**  **(N = 10)** | **EM**  **(N = 8)** | **CM**  **(N = 7)** | **Adj. *P* Value** | | | **HC**  **(N = 10)** | **EM**  **(N = 8)** | **CM**  **(N = 7)** | **Adj. *P* Value** | | |
|  |  |  |  | **HC-EM** | **HC-CM** | **EM-CM** |  |  |  | **HC-EM** | **HC-CM** | **EM-CM** |
|  | **LEFT side** | | | | | | **RIGHT side** | | | | | |
| Frontal Areas |  |  |  |  |  |  |  |  |  |  |  |  |
| Caudal Anterior Cingulate | 2.698 (0.067) | 2.743 (0.079) | 2.633 (0.084) | 0.673 | 0.553 | 0.362 | 2.488 (0.070) | 2.464 (0.083) | 2.567 (0.089) | 0.831 | 0.488 | 0.412 |
| **Caudal Middle Frontal** | 2.480 (0.054) | 2.481 (0.063) | 2.608 (0.069) | 0.986 | **0.028** | **0.043** | 2.414 (0.067) | 2.473 (0.080) | 2.578 (0.080) | 0.389 | **0.040** | 0.138 |
| Lateral Orbitofrontal | 2.664 (0.034) | 2.571 (0.041) | 2.668 (0.043) | 0.095 | 0.948 | 0.123 | 2.585 (0.039) | 2.494 (0.046) | 2.610 (0.049) | 0.151 | 0.688 | 0.105 |
| **Medial Orbitofrontal** | 2.317 (0.067) | 2.378 (0.057) | 2.472 (0.072) | 0.377 | **0.025** | 0.093 | 2.330 (0.049) | 2.305 (0.059) | 2.367 (0.062) | 0.748 | 0.642 | 0.480 |
| Paracentral | 2.397 (0.041) | 2.353 (0.049) | 2.383 (0.052) | 0.506 | 0.843 | 0.682 | 2.389 (0.038) | 2.399 (0.045) | 2.463 (0.048) | 0.865 | 0.244 | 0.356 |
| **Pars Opercularis** | 2.540 (0.040) | 2.596 (0.048) | 2.609 (0.041) | 0.386 | 0.302 | 0.857 | 2.638 (0.039) | 2.469 (0.047) | 2.604 (0.050) | **0.011** | 0.587 | 0.064 |
| Pars Orbitalis | 2.773 (0.060) | 2.615 (0.071) | 2.773 (0.075) | 0.103 | 0.996 | 0.144 | 2.747 (0.065) | 2.593 (0.078) | 2.722 (0.083) | 0.144 | 0.811 | 0.274 |
| Pars Triangularis | 2.417 (0.037) | 2.399 (0.044) | 2.465 (0.047) | 0.764 | 0.431 | 0.327 | 2.435 (0.045) | 2.431 (0.054) | 2.474 (0.057) | 0.957 | 0.593 | 0.592 |
| Precentral | 2.608 (0.036) | 2.562 (0.043) | 2.617 (0.046) | 0.427 | 0.873 | 0.396 | 2.533 (0.037) | 2.491 (0.044) | 2.592 (0.047) | 0.474 | 0.327 | 0.132 |
| Rostral Anterior Cingulate | 2.909 (0.058) | 2.856 (0.069) | 2.986 (0.074) | 0.370 | 0.420 | 0.132 | 2.743 (0.048) | 2.686 (0.057) | 2.783 (0.061) | 0.455 | 0.610 | 0.263 |
| **Rostral Middle Frontal** | 2.305 (0.043) | 2.287 (0.050) | 2.415 (0.054) | 0.688 | **0.022** | **0.016** | 2.186 (0.044) | 2.217 (0.054) | 2.316 (0.058) | 0.508 | **0.022** | 0.052 |
| **Superior Frontal** | 2.673 (0.052) | 2.684 (0.062) | 2.793 (0.067) | 0.836 | **0.028** | 0.062 | 2.630 (0.031) | 2.577 (0.037) | 2.707 (0.039) | 0.286 | 0.136 | **0.026** |
| Frontal Pole | 2.758 (0.069) | 2.823 (0.082) | 2.832 (0.088) | 0.552 | 0.513 | 0.943 | 2.612 (0.076) | 2.745 (0.090) | 2.696 (0.096) | 0.272 | 0.494 | 0.720 |
| Temporal areas |  |  |  |  |  |  |  |  |  |  |  |  |
| Banks Superior Temporal Sulcus | 2.250 (0.052) | 2.582 (0.062) | 2.571 (0.066) | 0.456 | 0.546 | 0.912 | 2.606 (0.033) | 2.586 (0.039) | 2.607 (0.042) | 0.692 | 0.992 | 0.718 |
| Entorhinal | 3.475 (0.106) | 3.429 (0.126) | 3.340 (0.134) | 0.783 | 0.436 | 0.636 | 3.599 (0.135) | 3.494 (0.160) | 3.529 (0.171) | 0.622 | 0.749 | 0.884 |
| Fusiform | 2.781 (0.038) | 2.724 (0.046) | 2.773 (0.049) | 0.351 | 0.901 | 0.474 | 2.810 (0.034) | 2.716 (0.040) | 2.829 (0.043) | 0.091 | 0.731 | 0.073 |
| Superior Temporal | 2.892 (0.038) | 2.785 (0.045) | 2.832 (0.048) | 0.087 | 0.342 | 0.490 | 2.874 (0.043) | 2.808 (0.051) | 2.833 (0.054) | 0.336 | 0.561 | 0.744 |
| Middle Temporal | 2.931 (0.053) | 2.912 (0.063) | 2.967 (0.067) | 0.821 | 0.673 | 0.559 | 2.922 (0.035) | 2.871 (0.042) | 2.939 (0.044) | 0.367 | 0.758 | 0.283 |
| Inferior Temporal | 2.883 (0.043) | 2.810 (0.051) | 2.891 (0.054) | 0.286 | 0.916 | 0.297 | 2.871 (0.042) | 2.848 (0.050) | 2.825 (0.053) | 0.736 | 0.508 | 0.758 |
| **Transverse Temporal** | 2.451 (0.067) | 2.291 (0.080) | 2.469 (0.085) | 0.141 | 0.868 | 0.146 | 2.506 (0.058) | 2.318 (0.069) | 2.435 (0.073) | **0.049** | 0.460 | 0.260 |
| Isthmus of cingulate | 2.254 (0.063) | 2.603 (0.074) | 2.482 (0.079) | 0.427 | 0.684 | 0.286 | 2.377 (0.049) | 2.491 (0.058) | 2.466 (0.062) | 0.153 | 0.274 | 0.777 |
| **Parahippocampal** | 2.892 (0.087) | 2.776 (0.104) | 2.693 (0.110) | 0.405 | 0.172 | 0.595 | 2.944 (0.100) | 2.777 (0.115) | 2.705 (0.122) | 0.080 | **0.018** | 0.488 |
| **Temporal Pole** | 3.717 (0.082) | 3.620 (0.098) | 3.720 (0.104) | 0.459 | 0.983 | 0.498 | 4.021 (0.086) | 3.677 (0.103) | 3.997 (0.100) | **0.018** | 0.863 | 0.057 |
| Insula | 3.064 (0.044) | 3.063 (0.053) | 3.082 (0.056) | 0.986 | 0.801 | 0.806 | 3.049 (0.051) | 3.034 (0.061) | 3.082 (0.065) | 0.850 | 0.693 | 0.597 |
| Parietal Areas |  |  |  |  |  |  |  |  |  |  |  |  |
| Superior Parietal | 2.211 (0.030) | 2.177 (0.036) | 2.254 (0.038) | 0.477 | 0.385 | 0.161 | 2.204 (0.033) | 2.185 (0.039) | 2.234 (0.041) | 0.716 | 0.579 | 0.409 |
| **Inferior Parietal** | 2.414 (0.034) | 2.441 (0.040) | 2.478 (0.043) | 0.625 | 0.252 | 0.530 | 2.452 (0.050) | 2.465 (0.060) | 2.559 (0.052) | 0.787 | **0.048** | 0.108 |
| Postcentral | 2.164 (0.030) | 2.091 (0.036) | 2.137 (0.038) | 0.135 | 0.588 | 0.392 | 2.115 (0.021) | 2.074 (0.026) | 2.091 (0.027) | 0.235 | 0.485 | 0.670 |
| Posterior Cingulate | 2.487 (0.039) | 2.553 (0.047) | 2.484 (0.50) | 0.296 | 0.956 | 0.328 | 2.453 (0.046) | 2.454 (0.055) | 2.528 (0.059) | 0.982 | 0.323 | 0.375 |
| Precuneus | 2.369 (0.035) | 2.343 (0.042) | 2.353 (0.045) | 0.643 | 0.774 | 0.881 | 2.414 (0.031) | 2.388 (0.037) | 2.422 (0.039) | 0.595 | 0.864 | 0.531 |
| Supramarginal | 2.627 (0.041) | 2.531 (0.049) | 2.644 (0.052) | 0.150 | 0.806 | 0.135 | 2.617 (0.039) | 2.508 (0.047) | 2.618 (0.050) | 0.089 | 0.988 | 0.125 |
| Occipital Areas |  |  |  |  |  |  |  |  |  |  |  |  |
| Cuneus | 1.869 (0.034) | 1.819 (0.040) | 1.838 (0.042) | 0.346 | 0.576 | 0.741 | 1.911 (0.042) | 1.865 (0.050) | 1.841 (0.053) | 0.485 | 0.309 | 0.749 |
| Lateral Occipital | 2.242 (0.043) | 2.259 (0.051) | 2.250 (0.054) | 0.801 | 0.907 | 0.907 | 2.290 (0.038) | 2.303 (0.045) | 2.313 (0.047) | 0.828 | 0.703 | 0.876 |
| Lingual | 2.002 (0.029) | 1.980 (0.035) | 2.022 (0.037) | 0.624 | 0.674 | 0.0415 | 2.089 (0.029) | 2.009 (0.034) | 2.051 (0.036) | 0.085 | 0.414 | 0.412 |
| Pericalcarine | 1.613 (0.039) | 1.611 (0.046) | 1.689 (0.049) | 0.979 | 0.237 | 0.268 | 1.652 (0.049) | 1.554 (0.058) | 1.679 (0.062) | 0.215 | 0.734 | 0.162 |

Bold font in the *P* values column indicates statistically significant.

Significance assessed with ANCOVA modelling adjusted by patients’ age in order to find cortical thickness differences between healthy controls and migraine patients (EM and CM).

HC: healthy controls, EM: episodic migraine, CM: chronic migraine, SD: standard deviation; Adj. *P* value: adjusted *P* value (Bonferroni correction)

| **SUPPLEMENTARY TABLE 3. Local gyrification indexes differences between healthy controls and migraine subgroups (CM and EM)** | | | | | | | | |  | | | |
| --- | --- | --- | --- | --- | --- | --- | --- | --- | --- | --- | --- | --- |
| **Anatomical Areas**, mean (SD) | **HC**  **(N = 10)** | **EM**  **(N = 8)** | **CM**  **(N = 7)** | **Adj. *P* Value** | | | **HC**  **(N = 10)** | **EM**  **(N = 8)** | **CM**  **(N = 7)** | **Adj. *P* Value** | | |
|  |  |  |  | **HC-EM** | **HC-CM** | **EM-CM** |  |  |  | **HC-EM** | **HC-CM** | **EM-CM** |
|  | **LEFT side** | | | | | | **RIGHT side** | | | | | |
| Frontal Areas |  |  |  |  |  |  |  |  |  |  |  |  |
| Caudal Anterior Cingulate | 1.904 (0.021) | 1.946 (0.025) | 1.896 (0.027) | 0.211 | 0.835 | 0.196 | 1.952 (0.026) | 2.006 (0.030) | 1.978 (0.032) | 0.187 | 0.538 | 0.530 |
| Caudal Middle Frontal | 3.116 (0.043) | 3.116 (0.051) | 3.063 (0.054) | 0.970 | 0.471 | 0.488 | 3.107 (0.045) | 3.076 (0.051) | 3.027 (0.054) | 0.651 | 0.265 | 0.516 |
| Lateral Orbitofrontal | 2.572 (0.033) | 2.611 (0.040) | 2.608 (0.042) | 0.462 | 0.516 | 0.955 | 2.570 (0.029) | 2.531 (0.032) | 2.571 (0.035) | 0.368 | 0.987 | 0.410 |
| Medial Orbitofrontal | 2.094 (0.021) | 2.061 (0.024) | 2.112 (0.026) | 0.315 | 0.604 | 0.178 | 2.176 (0.021) | 2.156 (0.024) | 2.193 (0.026) | 0.540 | 0.623 | 0.317 |
| Paracentral | 2.312 (0.028) | 2.282 (0.033) | 2.337 (0.035) | 0.499 | 0.583 | 0.275 | 2.357 (0.027) | 2.314 (0.030) | 2.303 (0.033) | 0.294 | 0.218 | 0.825 |
| Pars Opercularis | 4.155 (0.077) | 4.203 (0.092) | 3.974 (0.098) | 0.690 | 0.161 | 0.106 | 4.213 (0.093) | 4.073 (0.106) | 4.150 (0.113) | 0.331 | 0.674 | 0.626 |
| Pars Orbitalis | 2.929 (0.062) | 3.040 (0.074) | 2.969 (0.079) | 0.264 | 0.693 | 0.522 | 2.966 (0.048) | 2.902 (0.054) | 2.961 (0.058) | 0.386 | 0.948 | 0.472 |
| Pars Triangularis | 3.682 (0.070) | 3.755 (0.084) | 3.602 (0.089) | 0.516 | 0.487 | 0.231 | 3.747 (0.079) | 3.613 (0.089) | 3.679 (0.096) | 0.272 | 0.590 | 0.622 |
| Precentral | 3.386 (0.037) | 3.379 (0.044) | 3.305 (0.046) | 0.909 | 0.186 | 0.264 | 3.365 (0.048) | 3.319 (0.055) | 3.309 (0.059) | 0.531 | 0.467 | 0.905 |
| Rostral Anterior Cingulate | 2.039 (0.022) | 2.046 (0.026) | 2.045 (0.026) | 0.847 | 0.841 | 0.983 | 2.142 (0.024) | 2.145 (0.027) | 2.147 (0.029) | 0.932 | 0.877 | 0.946 |
| Rostral Middle Frontal | 2.735 (0.037) | 2.767 (0.044) | 2.664 (0.047) | 0.589 | 0.248 | 0.131 | 2.751 (0.033) | 2.773 (0.037) | 2.723 (0.040) | 0.660 | 0.598 | 0.377 |
| Superior Frontal | 2.201 (0.022) | 2.219 (0.027) | 2.175 (0.028) | 0.624 | 0.466 | 0.275 | 2.270 (0.020) | 2.277 (0.022) | 2.221 (0.024) | 0.805 | 0.133 | 0.106 |
| Frontal Pole | 2.065 (0.022) | 2.105 (0.026) | 2.066 (0.028) | 0.251 | 0.980 | 0.318 | 2.167 (0.022) | 2.187 (0.025) | 2.194 (026) | 0.564 | 0.437 | 0.831 |
| Temporal areas |  |  |  |  |  |  |  |  |  |  |  |  |
| Banks Superior Temporal Sulcus | 3.466 (0.056) | 3.413 (0.067) | 3.311 (0.071) | 0.552 | 0.099 | 0.311 | 3.546 (0.058) | 3.436 (0.066) | 3.387 (0.070) | 0.223 | 0.097 | 0.622 |
| Entorhinal | 2.487 (0.027) | 2.509 (0.032) | 2.491 (0.034) | 0.601 | 0.937 | 0.694 | 2.548 (0.037) | 2.552 (0.042) | 2.545 (0.045) | 0.947 | 0.950 | 0.906 |
| Fusiform | 2.609 (0.034) | 2.598 (0.041) | 2.573 (0.044) | 0.838 | 0.519 | 0.681 | 2.587 (0.031) | 2.552 (0.033) | 2.605 (0.038) | 0.456 | 0.724 | 0.321 |
| Superior Temporal | 3.942 (0.058) | 4.014 (0.069) | 3.879 (0.073) | 0.436 | 0.505 | 0.199 | 4.041 (0.068) | 3.917 (0.077) | 3.948 (0.082) | 0.240 | 0.395 | 0.787 |
| Middle Temporal | 3.280 (0.045) | 3.208 (0.053) | 3.172 (0.057) | 0.312 | 0.148 | 0.654 | 3.220 (0.051) | 3.192 (0.058) | 3.171 (0.062) | 0.723 | 0.552 | 0.810 |
| Inferior Temporal | 2.672 (0.035) | 2.659 (0.042) | 2.593 (0.045) | 0.813 | 0.179 | 0.302 | 2.593 (0.035) | 2.611 (0.040) | 2.647 (0.042) | 0.733 | 0.343 | 0.556 |
| Transverse Temporal | 4.503 (0.066) | 4.577 (0.078) | 4.398 (0.083) | 0.478 | 0.334 | 0.137 | 4.722 (0.082) | 4.533 (0.093) | 4.561 (0.099) | 0.142 | 0.226 | 0.841 |
| Isthmus of cingulate | 2.626 (0.048) | 2.663 (0.057) | 2.754 (0.061) | 0.627 | 0.110 | 0.289 | 2.839 (0.042) | 2.760 (0.048) | 2.742 (0.051) | 0.230 | 0.160 | 0.806 |
| Parahippocampal | 2.685 (0.032) | 2.683 (0.038) | 2.669 (0.041) | 0.966 | 0.759 | 0.807 | 2.664 (0.040) | 2.648 (0.046) | 2.694 (0.049) | 0.792 | 0.647 | 0.507 |
| Temporal Pole | 2.367 (0.039) | 2.411 (0.046) | 2.435 (0.049) | 0.475 | 0.284 | 0.722 | 2.404 (0.047) | 2.431 (0.053) | 2.450 (0.057) | 0.706 | 0.533 | 0.805 |
| Insula | 4.116 (0.077) | 4.107 (0.091) | 4.040 (0.097) | 0.939 | 0.542 | 0.624 | 4.252 (0.090) | 4.073 (0.102) | 4.212 (0.109) | 0.200 | 0.778 | 0.368 |
| Parietal Areas |  |  |  |  |  |  |  |  |  |  |  |  |
| Superior Parietal | 2.905 (0.042) | 2.949 (0.050) | 2.929 (0.053) | 0.508 | 0.730 | 0.785 | 2.894 (0.038) | 2.903 (0.042) | 2.869 (0.046) | 0.877 | 0.677 | 0.597 |
| Inferior Parietal | 3.155 (0.050) | 3.225 (0.060) | 3.110 (0.063) | 0.381 | 0.586 | 0.207 | 3.153 (0.043) | 3.139 (0.048) | 3.051 (0.052) | 0.834 | 0.142 | 0.230 |
| Postcentral | 3.420 (0.035) | 3.450 (0.042) | 3.372 (0.045) | 0.594 | 0.414 | 0.228 | 3.425 (0.050) | 3.407 (0.056) | 3.360 (0.061) | 0.810 | 0.413 | 0.579 |
| Posterior Cingulate | 2.171 (0.035) | 2.190 (0.042) | 2.209 (0.045) | 0.731 | 0.509 | 0.763 | 2.210 (0.029) | 2.203 (0.033) | 2.161 (0.035) | 0.882 | 0.292 | 0.391 |
| Precuneus | 2.839 (0.052) | 2.846 (0.062) | 2.818 (0.066) | 0.930 | 0.806 | 0.762 | 3.053 (0.046) | 2.963 (0.052) | 2.927 (0.056) | 0.209 | 0.095 | 0.642 |
| Supramarginal | 3.481 (0.045) | 3.589 (0.054) | 3.487 (0.057) | 0.139 | 0.930 | 0.214 | 3.544 (0.051) | 3.523 (0.058) | 3.465 (0.062) | 0.787 | 0.337 | 0.507 |
| Occipital Areas |  |  |  |  |  |  |  |  |  |  |  |  |
| Cuneus | 2.923 (0.054) | 2.903 (0.064) | 2.819 (0.068) | 0.811 | 0.244 | 0.388 | 3.118 (0.048) | 2.988 (0.054) | 2.976 (0.058) | 0.083 | 0.070 | 0.882 |
| Lateral Occipital | 2.597 (0.034) | 2.522 (0.040) | 2.522 (0.043) | 0.167 | 0.185 | 0.991 | 2.644 (0.038) | 2.542 (0.043) | 2.558 (0.046) | 0.086 | 0.160 | 0.801 |
| Lingual | 2.780 (0.043) | 2.739 (0.052) | 2.729 (0.055) | 0.553 | 0.473 | 0.896 | 2.892 (0.042) | 2.786 (0.048) | 2.805 (0.051) | 0.109 | 0.202 | 0.788 |
| Pericalcarine | 2.846 (0.058) | 2.796 (0.069) | 2.718 (0.073) | 0.585 | 0.182 | 0.451 | 3.001 (0.051) | 2.885 (0.058) | 2.895 (0.062) | 0.145 | 0.200 | 0.906 |

Significance assessed with ANCOVA modelling adjusted by patients’ age in order to find gyrification index differences between healthy controls and migraine patients (EM and CM).

HC: healthy controls, EM: episodic migraine, CM: chronic migraine, SD: standard deviation; Adj. *P* value: adjusted *P* value (Bonferroni correction
